# Decomposing complex reaction networks using random sampling, principal component analysis and basis rotation

**DOI:** 10.1186/1752-0509-3-30

**Published:** 2009-03-06

**Authors:** Christian L Barrett, Markus J Herrgard, Bernhard Palsson

**Affiliations:** 1 Department of Bioengineering; University of California at San Diego, La Jolla, CA, 92093-0412, USA; 2 Synthetic Genomics, 11149 North Torrey Pines Road, La Jolla, CA, 92037, USA

## Abstract

**Background:**

Metabolism and its regulation constitute a large fraction of the molecular activity within cells. The control of cellular metabolic state is mediated by numerous molecular mechanisms, which in effect position the metabolic network flux state at specific locations within a mathematically-definable steady-state flux space. Post-translational regulation constitutes a large class of these mechanisms, and decades of research indicate that achieving a network flux state through post-translational metabolic regulation is both a complex and complicated regulatory problem. No analysis method for the objective, top-down assessment of such regulation problems in large biochemical networks has been presented and demonstrated.

**Results:**

We show that the use of Monte Carlo sampling of the steady-state flux space of a cell-scale metabolic system in conjunction with Principal Component Analysis and eigenvector rotation results in a low-dimensional and biochemically interpretable decomposition of the steady flux states of the system. This decomposition comes in the form of a low number of small reaction sets whose flux variability accounts for nearly all of the flux variability in the entire system. This result indicates an underlying simplicity and implies that the regulation of a relatively low number of reaction sets can essentially determine the flux state of the entire network in the given growth environment.

**Conclusion:**

We demonstrate how our top-down analysis of networks can be used to determine key regulatory requirements independent of specific parameters and mechanisms. Our approach complements the reductionist approach to elucidation of regulatory mechanisms and facilitates the development of our understanding of global regulatory strategies in biological networks.

## Background

Metabolic network reconstructions[1] have been used as a basis for a number of analyses[2] that have provided insights into the topology [3-5], modularity[6,7], robustness[8], and dynamics[9] of large biochemical networks. In the constraint-based framework, the regulatory challenge for genome-scale metabolic networks has been described as a two-level process[10,11]: first, regulatory mechanisms associated with transcription and translation geometrically delimit the steady-state flux space by determining which reactions can potentially carry flux; and second, regulation of gene product activity by post-translational mechanisms determines the flux state as a point location within the flux space. The effective dimensionality of the first level of metabolic regulation has recently been shown to be small[12], but the effective dimensionality of the second level has yet to be assessed. We approach this problem of cell-scale post-translational regulation in the context of presenting a method for the decomposition of the range of functional capabilities of large biochemical reaction systems. We describe this decomposition procedure and demonstrate how it can elucidate a low number of reaction sets that account for nearly all of the range of behaviors in a cell-scale system.

## Results

**Figure 1:**
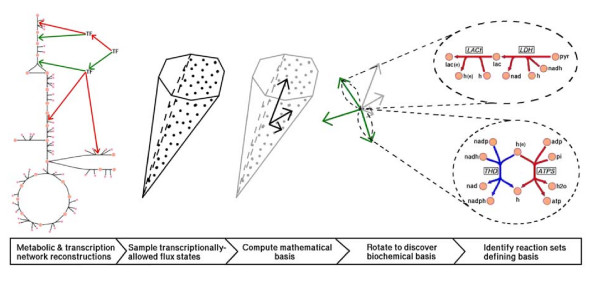
**The experimental procedure performed**. The possible steady-state flux states of the transcriptionally-allowed regions of the *E. coli *metabolic network are sampled and analyzed to reveal a small number of reaction sets that account for nearly all of the flux variation in a dynamic growth environment.

Our procedure is comprised of five main steps (Figure 1). The reconstructed integrated transcriptional regulatory and metabolic network of *E. coli *[13] was used for the analysis. We first defined a growth environment and then used the transcriptional regulatory network to determine which reactions could be active. This step corresponds to shrinking the flux space[10], and in effect reduces a 332-dimensional space to a 123-dimensional space. (Since a network flux distribution corresponds to a point location in the flux space, this dimensionality reduction result indicates that the post-translational regulatory challenge is approximately equal to or less than the transcriptional and translational challenge.) The environment simulated was glucose aerobic minimal media conditions in which all media components (i.e. oxygen, glucose, ammonia, sulfate, and phosphate) were allowed to vary from excess to limiting. The analysis described herein is equally applicable to different and more complex environments–in particular, the local environment that an organism is constantly altering (e.g diauxie.)

**Figure 2:**
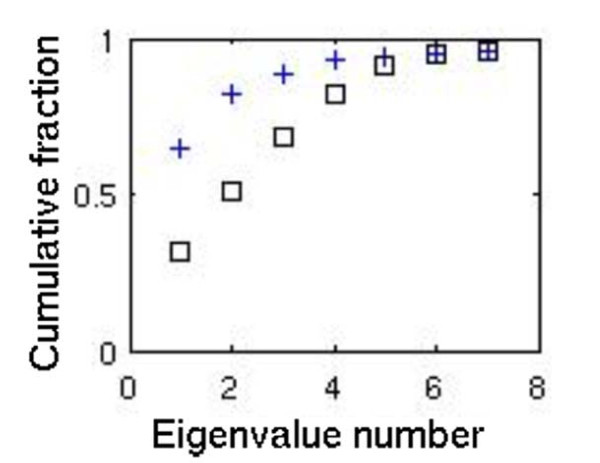
**The cumulative fractional eigenvalue distribution**. Shown for the variation in the randomly sampled metabolic network flux states before (crosses) and after (squares) eigenvector rotation.

Monte Carlo sampling is a method for generating large numbers of random allowable flux states and has been used to study the properties of metabolic flux states[9,14-19]. We comprehensively sampled the flux space corresponding to a growth rate of at least 90% of the maximum achievable growth rate and generated a large number (~10^6^) of flux vectors. Because the sampling procedure is a linear one and because we sought a basis for the sampled space, we performed Principal Components Analysis using Singular Value Decomposition. The cumulative fractional eigenvalue distribution (Figure 2) reveals that 96% of the variation in the metabolic network flux states can be explained by seven principal components–implying that the post-translational regulatory problem is low-dimensional. That is, by "regulating" a small number of dimensions the flux state of the entire network can be essentially set. The implications and caveats associated with this interpretation are addressed below.

A biochemically meaningful interpretation of the eigenvectors was found through the use basis rotation methods[20], which are able to minimize the ambiguous association between metabolic reactions and eigenvectors in an information-preserving manner. We rotated the top twenty eigenvectors and concentrated on the top seven (see Table 1 and Figure 2). We note three important results here. First, following rotation the eigenvectors were comprised of distinct sets of metabolic reactions. Second, all oblique and orthogonal rotation methods tested in this study produced very similar results, indicating that a natural structure was latent in the random flux samples. Third, we computed the correlation between all pairs of rotated eigenvectors and found them to have low correlation (see Additional file 1).

**Table 1:** The top twenty eigenfluxes resulting from the rotation procedure.

Eigenflux	Percent Variance	modality	Eigenflux description
1	34	1	Acetate overflow

2	20	2	Oxygen reduction

3	17	1	Glycolysis

4	14	2	Pyruvate overflow

5	9	2	NADPH from transhydrogenase

6	3	1	Lactate overflow

7	1	1	Ethanol overflow

8	0.99	1	TCA cycle

9	0.37	1	Ubiquinone reduction

10	0.36	1	NADH from soluble transhydrogenase

11	0.29	2	Anaplerotic use of phosphoenolpyruvate

12	0.13	1	Oxaloacetate from malate

13	0.13	1	Phosphopentomutase

14	0.13	2	Source of succinyl-CoA in TCA cycle

15	0.13	1	Adenine salvage

16	0.07	1	Glutamate synthesis

17	0.06	1	Ammonia transport

18	0.06	1	Inorganic pyrophosphatase

19	0.03	1	Pyruvate kinase

20	0.03	1	AMP recycling

**Figure 3:**
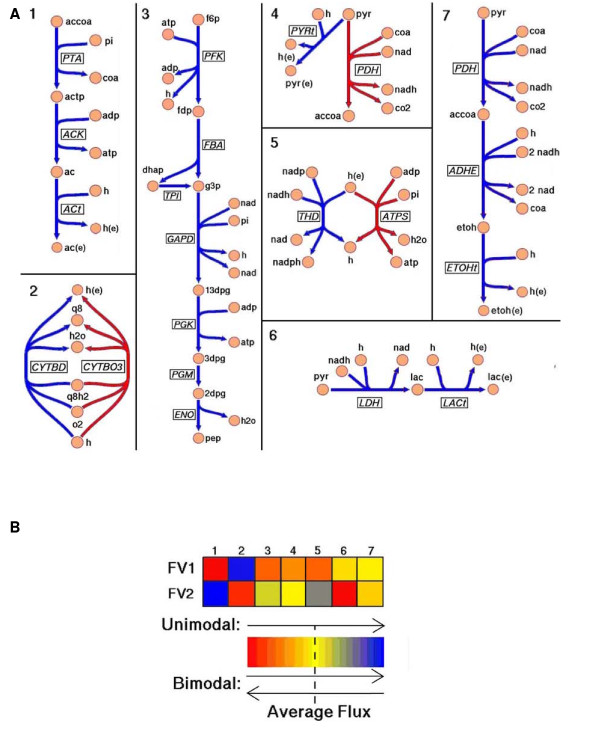
**Demonstration results of the described procedure in Figure 1**. A) The unimodal and bimodal reaction sets whose flux states essentially dictate the flux state of the entire metabolic network in glucose aerobic conditions. The reaction sets are colored to distinguish modality (blue and red for bimodal, blue for unimodal.) Extracellular metabolites are denoted with an appended '(e)'. See Additional file 4 for full metabolite and reaction names. B) The two randomly generated network flux distributions that most oppositely utilized the first reaction set in A), where the β values from Equation 1 are colored according to the accompanying color spectrum key.

We can interpret these independently-operable reaction sets, or eigenfluxes, as representing the regulatory challenge from a network perspective. Setting the seven eigenfluxes essentially positions the network flux state as a point within the flux space, and thus represents the second level of the two-level regulatory challenge[10,11]. The reaction loadings of the reactions on each eigenflux are all positive or are both positive and negative–indicating whether reactions in a set operate in a correlated (unimodal) or anti-correlated (bimodal) fashion. The reactions that comprise each eigenflux are illustrated in Figure 3A.

The reaction sets in Figure 3A are the flux altering mechanisms utilized by the *E. coli *model to maintain high biomass formation in the varying environment studied. (See Additional file 2 for the corresponding analysis for glucose anaerobic conditions.) These reaction sets can be examined in a biochemical and metabolic context:

1. Eigenfluxes 1, 4, 6, and 7, which together account for 49% of the flux variation, are metabolic "overflows." Overflow behavior in this context is due to oxygen limitation, and it allows the cell to generate ATP via substrate level phosphorylation and to oxidize NADH into NAD–which is in high demand. Both of these overflow-enabled mechanisms allow the cell to grow rapidly when glucose is in excess. Having a variety of overflow mechanisms allows the cell to balance its requirement to replenish NAD with its need to produce energy. The fluxes through these reactions are, in part, controlled by allosteric mechanisms. Phosphate acetyltransferase (PTA), which catalyzes the first reaction step of acetate secretion in eigenflux 1, is allosterically activated by pyruvate and inhibited by NADH and NADPH. High concentrations of NADH are indicative of a redox imbalance, which eigenfluxes 6 and 7 serve to correct by oxidizing NADH.

2. Eigenflux 2 is associated with establishing the proton motive force. By flexibly tuning the electro-chemical gradient of protons across the cellular membrane through translocation in the electron transport chain, a cell can economically provide the energy for essentially all cellular activity–such as ATP synthesis, solute transport, and flagellar motility. The cytochrome oxidases that catalyze the two opposing reactions in bimodal eigenflux 2 have slightly different functional behaviors. The cytochrome bo_3_oxidase (CYTBO3) is utilized under high oxygen concentrations and has a higher bioenergetic efficiency (protons translocated per electron) than the other cytochrome *bd *oxidase (CYTBD). Even though cytochrome *bd *is energetically less efficient it is operational at low oxygen levels.

3. Eigenflux 3 is the flux variation through glycolysis, and functions with eigenflux 5 to determine the absolute magnitudes of the fluxes in glycolysis and the pentose phosphate pathway (PPP).

4. Eigenflux 5 is associated with the dominant tradeoff in central metabolism for providing the metabolic precursors, ATP, and reducing power (in the form of NADPH) to generate macromolecular building blocks against the need for reducing power (in the form of NADH) for sustaining the proton gradient. Until recently it was assumed that nearly all of the NADPH needed for the biosynthesis reactions was produced in the PPP and the TCA cycle, but recent experiments[21] have demonstrated that in glucose aerobic conditions the proton-coupled transhydrogenase reaction provides 35%–45% of the NADPH needed for biosynthesis. Usage of the blue reaction in eigenflux 5 is consistent with this finding. Allosteric mechanisms at least in part mediate the balance between how the proton gradient is utilized and how flux is split between glycolysis and the PPP; the enzyme glucose-6-phophate-1-dehydrogenase, which catalyzes the first reaction of the PPP, is allosterically inhibited by NADH.

The flux carried through eigenfluxes in a given environment will be a result of molecular regulatory mechanisms, including mass action kinetics. The contribution of an eigenflux to any given steady-state flux vector **ν **can be calculated from the equation

(1) **β **= **U**^T ^* (**ν **- <**ν >**),

where **U **is the matrix of eigenfluxes and <**ν > **is the vector of mean flux values (see Additional file 3) as computed from the set of random flux samples. As can be seen from the inverse equation

(2) **ν **= <**ν **> + U* β,

the β values can be viewed as "tuning," or biasing, parameters–each β defining how the flux values for the reactions in its associated eigenflux are biased from their mean values. Thus one can effectively determine the flux state of the entire network with a low number of continuously varying and readily interpretable parameters.

To illustrate, we identified the two flux distributions that most oppositely utilized eigenflux 1 and computed their respective **β **vectors using Equation 1. The two selected flux distributions (FV1 and FV2) are presented as colored **β **vectors in Figure 3B. The color coding allows one to quickly identify that, while FV1 is secreting no acetate and is utilizing the cytochrome oxidase that translocates fewer protons (CYTBD), FV2 is maximally excreting acetate and almost exclusively utilizing the other cytochrome oxidase. Furthermore, FV1 is predominantly utilizing the proton gradient for ATP synthesis, while FV2 is utilizing it more for converting NADH to NADPH. The lower glycolytic flux of FV1 indicates a higher PPP flux and commensurately higher production of NADPH, which allows the proton gradient to be used more for ATP synthesis instead of NADPH production. The remaining components of **β **can be similarly interpreted, and altogether allow one to assess the flux state of the entire metabolic network at essentially a glance.

## Discussion

The faithfulness of the computational results of the presented procedure to biological reality depends critically on the completeness and integration of the molecular system reconstructions, assumptions made in the transformation of the integrated reconstructions into a model, and the manner in which the range of the system's functional capabilities are sampled. Cells are more than a combination of transcriptional regulatory and metabolic systems, so the exclusion of other cellular systems limits the scope of the decomposition that can be performed–as does the completeness of the included systems. For instance, with other systems included, the decomposition procedure could potentially identify osmotic or movement (i.e., flagellar) mechanisms in addition to purely metabolic mechanisms. Similarly, more complete regulatory information would allow a more accurate setting of reactions that can potentially carry flux. The fixed biomass composition in the utilized model does not accurately describe a cell in all growth environments for all growth rates. Such a fixed composition limits the exploration of how the relative amounts of the biomass components can vary or how the cell can utilize different operating regimes[22]. The range of behaviors of large, interacting systems of molecules is defined by high-dimensional mathematical spaces that are non-trivial to fully explore. Monte Carlo sampling of such spaces is not a solved problem, so the extent to which the full range of system capabilities can be sampled is directly related to the extent to which high-dimensional spaces can be computationally interrogated. While these issues are important caveats, they are also the subject of active research and so will gradually diminish in their limiting roles.

Molecular network reconstructions enable the objective, top-down assessment of regulatory challenges and functional capabilities associated with particular phenotypic states. In the context of metabolism, the method presented herein is also applicable to kinetic and free energy parameter spaces and to concentration space [23-26]. In general, it is applicable to any reconstructed cellular network in any environment and will aid in identifying (integrated) network regulatory challenges without the need to know detailed mechanisms or numerical parameter values.

Our results also shed light on the network topology-function relationship, which is the result of a lengthy evolutionary process in varying environments. Robustness[27,28], defined as the ability to maintain specific functions in the face of varying environmental conditions, is believed to constitute a primary determinant of the topology-function relationship. Since this relationship is manifested in the range of flux states that the network can support, our investigation illuminates how the network topology confers the ability to robustly maintain a high growth rate in a dynamic environment through the use of a small number of reaction mechanisms.

Similarly, the demonstrated procedure can be used to elucidate potential evolutionary mechanisms. By modifying the sampling procedure it is possible to simulate the evolution (i.e., increased substrate uptake and growth rate, increased fitness) of an organism in a particular growth environment. The decomposition procedure would identify the flux adjustment routes (i.e. eigenfluxes) by which evolution would be achieved.

## Conclusion

With a top-down view comes an understanding of what must be controlled to attain a network flux state, and with a reductionist view comes an understanding of the mechanisms that achieve such control. The top-down view will provide a context for the seemingly overlapping and redundant regulatory strategies that must make sense in not one environment, but in a large and varied range of environments. The analysis method presented here will help bridge the systems and molecular biology approaches for understanding cellular regulation in the context of large biochemical networks.

## Methods

### The model

We utilized integrated transcriptional regulatory and metabolic reconstruction iMC1010^v1 ^[13] for this study instead of the more current *E. coli *reconstruction because there are still unresolved issues with infeasible reaction cycles when performing Monte Carlo sampling. The iMC1010^v1 ^reconstruction is composed of 906 genes supporting 931 reactions (93% of which have been experimentally validated[29]) involving 625 metabolites, and includes 104 transcription factors regulating 470 of the 1,1010 total ORFs. The reconstruction is transformed into a model by the imposition of physical constraints. Oxygen and glucose uptake rates were constrained to be 15–20 mmol g/Dw/hr. Maximum ammonia, sulfate, and phosphate uptake rates were set based on the maximum needed for any oxygen and glucose uptake rate combination. Random uptake rates (see below) within these ranges constituted the varying growth environment simulated in this work. The minimum value for the "Biomass" demand reaction was set to be 90% of the maximal biomass that could be supported in the defined conditions. This allowable range of biomass generation constituted the high growth rate towards which robustness was assessed in this work.

### Preparation of the metabolic model for sampling

The model was utilized in this work to comprehensibly compute the range of flux states that the network can support in aerobic glucose minimal media conditions. The regulation included in the model plus the EcoCyc [30] and RegulonDB [31]databases were utilized to determine which reactions were "on", and could thus carry flux, in aerobic glucose conditions. Those reactions determined to be "off" were given reaction rate lower and upper bound values of zero. Additionally, all transport reactions for metabolites not in the defined minimal media were set such that their import flux was zero. All export fluxes were left unconstrained.

### Monte Carlo sampling

The COBRA Toolbox [32] for Matlab was utilized for most of the remaining steps, and functions mentioned below are from this toolbox.

Metabolic models contain reaction cycles that are responsible for some thermodynamically-impossible fluxes in computed model-wide flux distributions. These reaction cycles are dealt with in Energy Balance Analysis (EBA) [33] and can be easily identified as Type III pathways [34]. To remove the effect of such infeasible reaction cycles in the sampling procedure, we identified the Type III pathways and eliminated most of them by grouping cycle reactions together into a single "metareaction." This was accomplished with the function prepareForSampling with the parameters all 'true'. The few Type III pathways that could not be so eliminated were dealt during processing of the covariance matrix in the manner described below.

For sampling we used a modified version of an existing sampling algorithm [35,36] that has been previously applied to metabolism [9]–but with some noteworthy differences. Due to the very high dimensional, non-isotropic nature of the convex solution space, the generated samples from the standard algorithm are nearly all physiologically unrealistic by being very inefficient flux states characterized by high substrate uptake rates and very low growth rates. To correct for these issues, we biased the sampling algorithm to move towards higher growth rates. A Matlab m-file for this sampling algorithm is available on request. This sampling algorithm was used to generate 960,000 random network flux distributions with uptake and "Biomass" fluxes within the constrained ranges. Additional sampling did not change the marginal flux distributions–indicating that the flux space had been comprehensively sampled.

### Covariance matrix

The covariance matrix for reaction fluxes was calculated in the standard way [37] by first standardizing flux values with mean subtraction and then computing the covariance between all pairs of reactions. Those reactions involved in thermodynamically-infeasible reaction cycles (Type III pathways) that could not be grouped were eliminated from the covariance matrix. In practice this involved on the order of 1% of all reactions. As many reactions demonstrated very little variance, further analysis and interpretation of the covariance matrix was aided by removing such reactions. We removed any reaction from the covariance matrix if its variance was less than 1/80 th the value of the largest variance in the matrix. In effect, this additional processing removed 3.5% of the total system variance from the covariance matrix.

### Reporting Eigenvectors

The Singular Value Decomposition [38] was used to compute the eigenvectors and eigenvalues of the covariance matrix. The top twenty of these, explaining 99.33% of the variance in the covariance matrix (or about 96% of the variance of covariance before low variance reactions were removed), were rotated using the orthogonal varimax rotation procedure [39]. In reporting the reactions whose loadings dominated or defined unrotated and rotated eigenvectors, we report any reaction whose loading value is at least half of the largest absolute loading value of the eigenvector.

### Rotation Methods

The oblique and orthogonal rotation methods tested were oblimin, promax, varimax, quartimax, parsimax, equimax, and orthomax [20].

## Authors' contributions

CLB conceptualized and developed the method and MJH assisted in the development process. CLB wrote the manuscript, and all authors participated in manuscript editing.

## Supplementary Material

Additional File 1 **Correlation between eigenfluxes**. A histogram of the correlation between eigenfluxes derived by SVD of the flux correlation matrix. Click here for file

Additional File 4 **Model components.** Two tables listing the full and abbreviated names of the metabolites and the reactions comprising the model used in this work. Click here for file

Additional File 2 **Results from application of presented procedure for glucose anaerobic conditions**. A figure showing the cumulative fractional eigenvalue spectrum of the eigenfluxes and a table describing the reactions in each eigenflux. Click here for file

Additional File 3 **Mean and variance of reaction rates computed from sampling data.** A table showing the mean and variance fluxes resulting from Monte Carlo sampling in glucose aerobic conditions. Click here for file
