# Supplementary material for: Decomposing complex reaction networks using random sampling, principal component analysis and basis rotation
Source: BMC Syst Biol. 2009 Mar 6;3:30. doi: 10.1186/1752-0509-3-30 (PMC2667477; doi:10.1186/1752-0509-3-30)

**Additional Figure 1:** A histogram of the correlation between eigenvectors of the covariance matrix obtained using the oblique promax rotation. The figure demonstrates that even with the orthogonal rotation constraint removed, the rotated eigenvectors (and so the unimodal and bimodal eigenfluxes that they identify) are nearly independent.


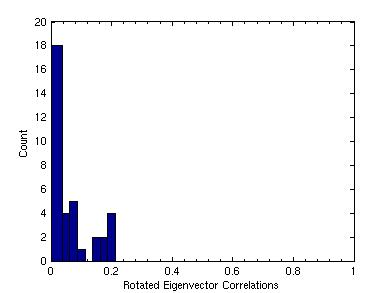

Supplement: Additional File 1 — Correlation between eigenfluxes. A histogram of the correlation between eigenfluxes derived by SVD of the flux correlation matrix. [file 1752-0509-3-30-S1.doc]
