# Supplementary material for: Decomposing complex reaction networks using random sampling, principal component analysis and basis rotation
Source: BMC Syst Biol. 2009 Mar 6;3:30. doi: 10.1186/1752-0509-3-30 (PMC2667477; doi:10.1186/1752-0509-3-30)
Supplement: Additional File 4 — Model components. Two tables listing the full and abbreviated names of the metabolites and the reactions comprising the model used in this work. [file 1752-0509-3-30-S4.doc]

Additional Table 3: Abbreviated and full metabolite names

| 13dpg | 3-Phospho-D-glyceroyl phosphate |
| --- | --- |
| 2dpg | D-glycerate 2-phosphate |
| 2dr1p | 2-Deoxy-D-ribose 1-phosphate |
| 2dr5p | 2-Deoxy-D-ribose 5-phosphate |
| 3dpg | 3-Phospho-D-glycerate |
| ac | Acetate |
| acald | Acetaldehyde |
| accoa | Acetyl-CoA |
| actp | Acetyl phosphate |
| ade | Adenine |
| adp | ADP |
| amp | AMP |
| atp | ATP |
| coa | Coenzyme A |
| dad2 | Deoxyadenosine |
| dadp | dADP |
| damp | dAMP |
| dha | Dihydroxyacetone |
| dhap | Dihydroxyacetone phosphate |
| etoh | Ethanol |
| f6p | D-Fructose 6-phosphate |
| fdp | D-Fructose 1,6-bisphosphate |
| g3p | Glyceraldehyde 3-phosphate |
| g6p | D-Glucose 6-phosphate |
| h | H+ |
| h2o | H2O |
| lac-D | D-Lactate |
| nad | Nicotinamide adenine dinucleotide |
| nadh | Nicotinamide adenine dinucleotide - reduced |
| nadp | Nicotinamide adenine dinucleotide phosphate |
| nadph | Nicotinamide adenine dinucleotide phosphate - reduced |
| o2 | O2 |
| pep | Phosphoenolpyruvate |
| pi | Phosphate |
| pyr | Pyruvate |
| q8 | Ubiquinone-8 |
| q8h2 | Ubiquinol-8 |
| r1p | alpha-D-Ribose 1-phosphate |
| r5p | alpha-D-Ribose 5-phosphate |
| ru5p-D | D-Ribulose 5-phosphate |
| trdox | Oxidized thioredoxin |
| trdrd | Reduced thioredoxin |

Additional Table 4: Abbreviated and full reaction names

| ACKr | acetate kinase |
| --- | --- |
| ACt | acetate reversible transport via proton symport |
| ADHE | Acetaldehyde dehydrogenase |
| ADK | adenylate kinase |
| ATPS | ATP synthase |
| CYTBD | cytochrome oxidase bd (ubiquinol-8) |
| CYTBO3 | cytochrome oxidase bo3 (ubiquinol-8) |
| D-LACt | D-lactate reversible transport via proton symport |
| DADK | deoxyadenylate kinase |
| DHAPT | Dihydroxyacetone phosphotransferase |
| DRPA | deoxyribose-phosphate aldolase |
| ENO | enolase |
| ETOHt | ethanol reversible transport via proton symport |
| F6PA | fructose 6-phosphate aldolase |
| FBA | fructose-bisphosphate aldolase |
| FORt | formate transport |
| G6PDH | glucose-6 phosphate dehydrogenase |
| GAPD | glyceraldehyde-3-phosphate dehydrogenase |
| LDH | D-lactate dehydrogenase |
| NADH5 | NADH dehydrogenase (ubiquinone-8, non proton tranlocating) |
| NADH6 | NADH dehydrogenase (ubiquinone-8, proton translocating) |
| NTD | 5'-nucleotidase (dAMP) |
| NTD2 | 5’-nucleotidase (UMP) |
| PDH | pyruvate dehydrogenase |
| PFK | phosphofructokinase |
| PFL | pyruvate formate lyase |
| PGL | 6-phosphogluconolactonase |
| PGI | glucose-6-phosphate isomerase |
| PGK | phosphoglycerate kinase |
| PGM | phosphoglycerate mutase |
| PPM | phosphopentomutase |
| PPM2 | phosphopentomutase 2 (deoxyribose) |
| PTA | phosphotransacetylase |
| PUNP | purine-nucleoside phosphorylase (Deoxyadenosine) |
| PYK | pyruvate kinase |
| PYRt | pyruvate reversible transport via proton symport |
| RNDR | ribonucleoside-diphosphate reductase (ADP) |
| RPI | ribose-5-phosphate isomerase |
| THD | NAD(P) transhydrogenase |
| TPI | triose-phosphate isomerase |
| TRDR | thioredoxin reductase (NADPH) |
| URIK | uridine kinase |
