# Supplementary material for: Decomposing complex reaction networks using random sampling, principal component analysis and basis rotation
Source: BMC Syst Biol. 2009 Mar 6;3:30. doi: 10.1186/1752-0509-3-30 (PMC2667477; doi:10.1186/1752-0509-3-30)
Supplement: Additional File 2 — Results from application of presented procedure for glucose anaerobic conditions. A figure showing the cumulative fractional eigenvalue spectrum of the eigenfluxes and a table describing the reactions in each eigenflux. [file 1752-0509-3-30-S2.doc]

**Additional Figure 2**: The cumulative fractional eigenvalue distribution for the reaction flux covariance matrix before (crosses) and after (squares) eigenvector rotation for glucose anaerobic environmental conditions. The eigenflux descriptions are described below in Additional Table 1.


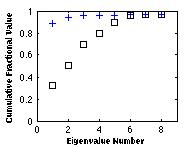


Additional Table 1: The first eight eigenfluxes for glucose anaerobic conditions.

| Fluxstat | Mode 1 Reactions | Mode 2 Reactions |
| --- | --- | --- |
| 1 | PTA, ACK, ACt |  |
| 2 | ATPS, THD |  |
| 3 | G6PDH, PGL, GND, | PGI |
| 4 | GAPD, PGK, PGM, ENO |  |
| 5 | PFL, FORt | PYK, ADHE, ETOHt |
| 6 | PFK, FBA, PYK | F6PA, DHAPT |
| 7 | NTD2, URIK |  |
| 8 | PYRt | LDH, LACt |
