# Supplementary material for: Decomposing complex reaction networks using random sampling, principal component analysis and basis rotation
Source: BMC Syst Biol. 2009 Mar 6;3:30. doi: 10.1186/1752-0509-3-30 (PMC2667477; doi:10.1186/1752-0509-3-30)
Supplement: Additional File 3 — Mean and variance of reaction rates computed from sampling data. A table showing the mean and variance fluxes resulting from Monte Carlo sampling in glucose aerobic conditions. [file 1752-0509-3-30-S3.doc]

Additional Table 2: Mean and variance of reaction rates computed from sampling data.

| **Reaction** | **Mean Flux** (sec^-1^) | **Flux Variance** (sec^-2^) |
| --- | --- | --- |
| ACK | 13.200 | 10.169 |
| ACt | 13.973 | 10.281 |
| ADHE | 2.216 | 0.583 |
| ATPS | 47.578 | 8.242 |
| CYTBD | 3.960 | 14.693 |
| CYTBO3 | 35.856 | 19.591 |
| D-LACt | 0.638 | 2.520 |
| DADK | 0.336 | 0.280 |
| DHAPT | 4.830 | 1.430 |
| DRPA | 0.691 | 0.639 |
| ENO | 30.602 | 2.152 |
| ETOHt | 2.216 | 0.583 |
| F6PA | 5.674 | 1.389 |
| FBA | 11.195 | 0.563 |
| GAPD | 33.162 | 2.035 |
| LDH | 0.685 | 2.683 |
| NADH5 | 0.032 | 0.793 |
| NADH6 | 38.985 | 3.632 |
| NTD | 0.342 | 0.279 |
| PDH | 21.601 | 11.739 |
| PFK | 11.246 | 0.582 |
| PGI | 17.002 | 5.904 |
| PGK | 33.162 | 2.035 |
| PGM | 30.602 | 2.152 |
| PPM | 0.066 | 1.276 |
| PPM2 | 0.691 | 0.639 |
| PTA | 13.200 | 10.169 |
| PUNP | 0.313 | 0.273 |
| PYRt | 0.257 | 4.939 |
| RNDR | 0.373 | 0.278 |
| RPI | 2.177 | 1.162 |
| THD | 16.567 | 16.480 |
| TPI | 15.785 | 1.021 |
| TRDR | 1.115 | 0.616 |
